# Birth of Four Children

**Published:** 1845-07

**Authors:** 


*Birth of Four Children.*—A healthy, strong woman, ætat 35, after having had five children, became pregnant again, and this time her abdomen appeared to be of larger dimensions than usual. During the subsequent process of parturition, the waters were discharged sparingly; one child presented itself with the feet, and was expelled by the efforts of nature. Half an hour after, the birth of the second child began and was terminated in an hour. The third and fourth children (apparently still-born) speedily followed. The quantity of water discharged was about 2lb. After one hour and three quarters, four separate placentæ were expelled. These four children (boys) were all born with the feet foremost; they were all perfectly mature and well formed; they resembled each other, and performed their functions regularly. Their length averaged from 15½ to 17 inches, their weight from 3lb. to 4lb. They all died within the next five days, notwithstanding the greatest care was taken of them as regards their nourishment and preservation. The mother left her bed five days after delivery, and remains in very good health.—*Lond. Med. Times, from Prof. Pfau in Oester. Medic. Wochenschr.*

